# Scale-Up of the Electrodeposition of ZnO/Eosin Y Hybrid Thin Films for the Fabrication of Flexible Dye-Sensitized Solar Cell Modules

**DOI:** 10.3390/ma11020232

**Published:** 2018-02-02

**Authors:** Florian Bittner, Torsten Oekermann, Michael Wark

**Affiliations:** 1 Institute of Physical Chemistry and Electrochemistry, Leibniz University Hannover, Callinstr. 3a, 30167 Hannover, Germany; florian.bittner@wki.fraunhofer.de (F.B.); torsten.oekermann@saftbatteries.com (T.O.); 2 Application Center for Wood Fiber Research HOFZET^®^, Fraunhofer Institute for Wood Research, Wilhelm-Klauditz-Institute WKI, Heisterbergallee 10A, 30453 Hannover, Germany; 3 Friemann & Wolf Batterietechnik GmbH, Industriestr. 22, 63654 Büdingen, Germany; 4 Institute of Chemistry, Chemical Technology 1, Carl von Ossietzky University Oldenburg, Carl-von-Ossietzky-Str. 9-11, 26129 Oldenburg, Germany

**Keywords:** electrochemical deposition, scale-up, zinc oxide, eosin Y, dye-sensitized solar cell, solar module

## Abstract

The low-temperature fabrication of flexible ZnO photo-anodes for dye-sensitized solar cells (DSSCs) by templated electrochemical deposition of films was performed in an enlarged and technical simplified deposition setup to demonstrate the feasibility of the scale-up of the deposition process. After extraction of eosin Y (EY) from the initially deposited ZnO/EY hybrid films, mesoporous ZnO films with an area of about 40 cm^2^ were reproducibly obtained on fluorine doped tin oxide (FTO)-glass as well as flexible indium tin oxide (ITO)–polyethylenterephthalate (PET) substrates. With a film thickness of up to 9 µm and a high specific surface area of up to about 77 m^2^·cm^−3^ the ZnO films on the flexible substrates show suitable properties for DSSCs. Operative flexible DSSC modules proved the suitability of the ZnO films for use as DSSC photo-anodes. Under a low light intensity of about 0.007 sun these modules achieved decent performance parameters with conversion efficiencies of up to 2.58%. With rising light intensity the performance parameters deteriorated, leading to conversion efficiencies below 1% at light intensities above 0.5 sun. The poor performance of the modules under high light intensities can be attributed to their high series resistances.

## 1. Introduction

Currently the generation of electricity by photovoltaics is almost completely limited to stationary solutions. Mobile applications, such as solar cells integrated in clothes for charging mobile electronic devices, have been largely unexploited, but promise huge potentials for environmental benefit and commercial success [1]. Flexible solar cells based on thin film technologies are especially suitable for mobile applications due to their low weight, their breaking resistance, and their adaptability. Furthermore, the manufacturing costs of thin film solar cells are potentially lower than those of conventional silicon-based solar cells. Roll-to-roll processes, which are applicable to flexible substrates, promise high production throughputs [1,2,3].

One of the thin film solar cell technologies suitable for the realization of flexible devices is that of dye-sensitized solar cells (DSSCs) [4,5]. The conversion efficiency record for DSSCs (reported for an active area of 0.36 cm^2^ in 2011) of 12.3% on glass-based substrates [6] is higher than that recently reported for organic solar cells (11.2% [7]) and a-Si solar cells (10.2% [7]). The conversion efficiencies of inorganic thin film solar cells, such as 21.7% [8] for cells based on copper indium gallium diselenide, have not been achieved, but lower production costs are predicted and a much less quantity of low abundant or toxic metals is needed [3]. Another advantage of DSSCs in comparison to other solar cell technologies is the better utilization of diffuse or low-intensity illumination. As unique characteristics, DSSCs are semi-transparent, if both electrodes are based on transparent substrate materials, and their color can be varied [2,3]. The semi-transparency permits a versatile optical design of the solar cells.

Flexible DSSCs have been demonstrated in different configurations. Conversion efficiencies of up to 8.6% [9] have been reported for solar cells with photo-anodes based on metal substrates, which enable the sintering of the nanoparticular TiO_2_ thin films [10,11,12,13]. Disadvantages of this configuration are the necessary back-side illumination that involves optical losses and the sacrifice of the semi-transparency of the solar cells. To preserve the semi-transparency, both electrodes have to be based on transparent plastic foil substrates. Most commonly used are foils of polyethylenterephthalate (PET) or polyethylennaphthalate (PEN) that are coated with indium tin oxide (ITO) as transparent conducting layer. Since plastic foils are not resistant against the high temperatures associated with the TiO_2_ sintering process, low-temperature methods for the photo-anode fabrication have to be applied [4,5,14]. Several methods such as pressing [15], friction transfer [16], chemical sintering [17,18], or electrophoretic deposition [14,19] have been suggested to fabricate TiO_2_ thin films on plastic foil substrates. A conversion efficiency of *η* = 7.6% for flexible DSSCs based on plastic foils has been reported using the pressing method for TiO_2_ films on ITO-PEN substrates, where a pressure of 100 MPa was applied [15].

Besides TiO_2_, ZnO has been evaluated as semiconductor material for DSSCs and organic perovskite based solar cells; with the organic perovskite CH_3_NH_3_PbI_3_ recently conversion efficiencies up to 15.4% have been reported [20]. It features a higher electron mobility than TiO_2_ and it can be synthesized easily at low temperatures in several morphologies and with a very high crystallinity [21,22]. Porous ZnO thin films obtained by electrochemical deposition with assistance of a structure directing agent (SDA) showed particularly favorable properties as DSSC photo-anode material such as fast electron transport [23]. The method utilizes the dye molecule eosin Y (EY) as SDA [24]. After removal of the SDA highly crystalline nanoporous ZnO remains. The temperature applied during film deposition is 70 °C, rendering the method feasible for application on plastic foil substrates. Using the indoline dye D149 as sensitizer, a conversion efficiency of 5.56% has been achieved with this kind of ZnO film on rigid glass substrates [24]. A comprehensive description of this deposition method is given by Yoshida et al. [24]. Briefly summarized, the deposition method is based on the cathodic reduction of dissolved oxygen in aqueous Zn^2+^ solutions, forming hydroxide ions. Zn^2+^ and OH^−^ ions precipitate on the substrate as Zn(OH)_2_, which rapidly dehydrates to ZnO at temperatures above 50 °C [24,25,26,27]:

O_2_ + 2H_2_O + 4e^−^ → 4OH^−^ (1)

Zn^2+^ + 2OH^−^ → Zn(OH)_2_ → ZnO + H_2_O (2)

By carrying out the first deposition step without addition of an SDA, a dense layer of compact and highly crystalline ZnO is formed that can act as blocking layer. In the assembled DSSC it is supposed to prevent recombination between electrons in the conductive substrate layer and tri-iodide ions in the electrolyte [24,26,27,28,29,30,31]. When EY is added to the deposition solution as an SDA, the dye accelerates the reduction of O_2_ and consequently the film growth. While both the non-reduced EY^2−^ and the electrochemically reduced EY^4−^ ions can be incorporated into the growing ZnO film, only the latter influences the morphology of the ZnO film significantly, resulting in a nanostructured hybrid film [24,30,32,33]. Therefore, the film deposition has to be performed at a potential more negative than the reduction potential of EY^2−^ (ca. −0.8 V vs. Ag/AgCl). The EY can be removed from the hybrid film by alkaline treatment, leaving a highly crystalline and porous ZnO structure [24,33,34,35].

For the electrodeposition of small-scaled ZnO films, rotating disc electrodes (RDEs) have been routinely used [24,36], since they provide fast and homogeneous mass transfer in the solution towards the electrode surface. This is a prerequisite for the preparation of uniform films. Since the technically complex RDE is limited to a deposition area of a few square centimeters, the suitability for scale-up of the deposition method has not yet been demonstrated. For this purpose we have developed a technically simplified electrodeposition setup that allows the deposition of ZnO/EY hybrid films on substrates up to about 60 cm^2^. Being sized between the small laboratory scale and the pilot plant scale, the setup is referred to as a miniplant setup.

This paper presents a detailed description of the miniplant setup and its use for the fabrication of porous ZnO films on glass and plastic foil substrates. The structure of the deposited ZnO films has been compared to that of films prepared in an RDE setup and optimized by adjustment of deposition parameters such as deposition time, electrode potential, and substrate layout. To illustrate the suitability of the obtained ZnO electrodes for the use in flexible DSSCs, solar cell module demonstrators were prepared and characterized.

## 2. Experimental Methods

### 2.1. Electrodeposition

#### 2.1.1. General Procedures

The electrolyte composition and conditions for the electrodeposition of ZnO and ZnO/EY hybrid films were based on the commonly used procedure [24,37]. Aqueous solutions of KCl (*c* = 0.1 mol∙L^−1^, ≥99.5%, Carl Roth, Karlsruhe, Germany), ZnCl_2_ (*c* = 5 mmol∙L^−1^, ≥98%, ABCR, Karlsruhe, Germany) and, in the case of the deposition of hybrid films, additionally eosin Y (*c* = 80 µmol∙L^−1^, ≥80%, Acros Organics, Geel, Belgium) were used as electrolyte. Oxygen saturation of the solutions was achieved by gas bubbling. The solutions were kept at a temperature of 70 °C. Fluorine doped tin oxide (FTO)-coated glass (7 Ω·sq^−1^, Pilkington TEC A8, NSG group, Minato-ku, Tokyo, Japan) or ITO-coated PET foil (50 Ω·sq^−1^, CPFilms OC™50, or 15 Ω·sq^−1^, CPFilms LR15, Canoga Park, CA, USA) served as substrate material. FTO-glass substrates and ITO-PET foil substrates LR15 were pre-treated in a mildly alkaline cleaning agent (1% solution of deconex^®^ 12 BASIC, Borer Chemie AG, Zuchwil, Switzerland).

#### 2.1.2. Fabrication of Small-Scaled ZnO Films

The electrodeposition of small-scaled ZnO and ZnO/EY hybrid films was performed on an RDE (Metrohm Autolab RDE-2, Metrohm Autolab, Utrecht, The Netherlands), rotating with 300 rpm. The deposition area was fixed to 1.54 cm^2^. A zinc wire (*d* = 1 mm, ≥99.95%, Thermo Fisher (Kandel) GmbH, Karlsruhe, Germany) was used as the counter electrode. The deposition was performed at −0.91 V vs. Ag/AgCl. Compact ZnO films were deposited for 10 min, ZnO/EY hybrid films on top of the compact ZnO films were deposited for 45 min. The desorption of the EY was achieved by immersing the films for 24 h in an aqueous solution of KOH (*V* = 500 mL, pH = 10.5, ≥85%, Applichem, Darmstadt, Germany).

#### 2.1.3. Up-Scaled Fabrication of ZnO Films

**Figure 1:**
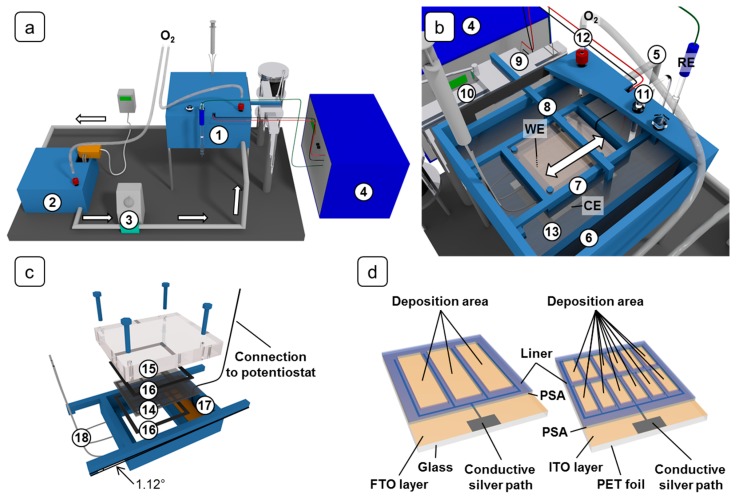
Schematic illustration of the miniplant setup. (**a**) Total view: (1) Deposition basin, (2) Tempering basin, (3) Diaphragm pump, (4) Potentiostat. (**b**) Deposition basin: (5) Inlet, (6) Overflow, (7) Substrate holder, (8) Paddle, (9) Slide, (10) Linear guide, (11) Salt bridge, (12) Glass frit, (13) Zinc foil. (**c**) Substrate holder: (14) Substrate, (15) pressure plate, (16) sealing mats, (17) connection area, (18) gas suction tubes. (**d**) Layout of the FTO-glass (left) and ITO-PET (right) substrates.

The miniplant setup that was developed for the up-scaled electrodeposition is schematically illustrated in Figure 1a. It consists of a deposition basin (1) including a substrate holder and a motor-driven paddle, a tempering basin (2), a diaphragm pump (3), and a potentiostat (4). Polypropylene was used as construction material for the basins. The electrodes are connected to the potentiostat (Amel Instruments Mod. 7050, Milan, Italy) in a 3-electrode-setup. The pump (Liquiport NF300KT.18S, KNF Neuberger, Balterswil, Switzerland) forwards the deposition solution from the tempering basin to the deposition basin, from where it flows back into the tempering basin. A flow rate of 2.21 L·min^−1^ was determined as optimum setting of the diaphragm pump, giving a satisfactory electrolyte flow while keeping vibrations low. The volume of the solution necessary for the electrodeposition is approximately 6.5 L.

To heat the solution, the basin contains a bended heating element encapsulated in polytetrafluoroethylene (PTFE) (custom build Nuega PTFE heating element, 800 W, 13 mm diameter and 1300 mm length) and a combined temperature/liquid level probe (custom build Nuega PTFE/graphite probes, Nuega, Georgensgmuend, Germany).

Figure 1b depicts the deposition basin. The deposition solution is pumped into the basin via the inlet (5) and flows across an overflow (6) back into the tempering basin (2). The overflow is necessary to maintain a constant level of the solution.

During electrodeposition the substrate in the substrate holder (7) serves as the working electrode (WE) and is immersed into the solution with the conductive side pointing downwards. The substrate holder is described below in more detail.

To ensure a homogeneous convection of the solution, a paddle (8) performs a forward and backward movement underneath the substrate. For this purpose the paddle is attached to a slide (9) on a linear guide (10). The surface of the paddle in the solution has dimensions of about 15.5 × 2.5 mm^2^. The movement of the slide is promoted by an electric motor with planetary gearhead via an eccentric disc. The operation of the electric motor at its power limit results in 42.5 cycles per minute for the paddle movement.

A salt bridge (11) (KCl, *c* = 0.1 mol∙L^−1^) connected to an Ag/AgCl reference electrode (RE, XR300, Radiometer analytical, Lyon, France) and a glass frit (12) for oxygen influx are placed in the solution. A zinc foil (13) (ABCR, ≥99.9%, thickness 0.62 mm) with dimensions of 150 × 100 mm^2^ acts as counter electrode (CE). The WE and CE wires, which are in contact with the deposition solution, are sheathed with PTFE.

The design of the substrate holder is depicted in Figure 1c. A cut-out of 75 × 75 mm^2^ allows the contact of the substrate with the deposition solution. The substrate (14) is pressed onto the base plate through a pressure plate (15). Tailored sealing mats (16) (EPDM 65, thickness 1.5 mm, Eriks NordOst, Hannover, Germany) on both sides of the substrate ensure a tight contact between substrate and base plate. The substrate is connected to the potentiostat on the end (17) which is not covered by the sealing mats and which features a dedicated area coated with conductive silver. The WE cable is soldered on a copper plate, against which the substrate is pressed. Additionally, conductive copper tape promotes electrical contact between the substrate and the copper plate. To remove gas bubbles underneath the substrate, the substrate holder has a tilt of 1.12° to collect the gas bubbles on one side of the cut-out. Via three PTFE tubes (18), which are incorporated into the substrate holder, the gas is sucked off before starting the electrodepositions and, if the deposition duration exceeds 20 min, during the deposition as well.

As illustrated in Figure 1d, the substrates are equipped with conducting silver paths to lower the ohmic voltage drop. On FTO-glass substrates the silver paths were prepared manually with silver paste (Acheson Silver DAG 1415, Agar Scientific, Essex, UK), on ITO-PET substrates they were applied by screen printing (mesh: PET 120-34 Y; silver paste: InkTec TEC-PA-040, Ansan-city, Kyungki-do, South Korea). To protect the silver from contact with the deposition solution (and eventually with the redox electrolyte in the DSSC), the paths are masked with tailored pressure-sensitive adhesives (PSA) (tesa^®^ 61562, Hamburg, Germany). At the same time, the PSAs served as spacers between the two electrodes in the assembled DSSCs. During the deposition process the PSAs were protected with liners.

Details about the implications of the electrodeposition scale-up—influencing the substrate layout and the deposition procedure—are given in the results Section 3.1.1.

To ensure a sufficient oxygen content of the solution the depositions were started not earlier than 15 min after the substrate holder was inserted into the deposition basin. The oxygen content prior to the deposition was determined with a colorimetric test kit (Macherey-Nagel VISOCOLOR^®^ ECO Oxygen, Dueren, Germany), proving saturation with oxygen. Directly after the deposition of ZnO/EY hybrid films the substrates were immersed in an aqueous KOH solution (*V* = 3 L, pH = 11, ≥85%, Applichem, Darmstadt, Germany) to desorb the EY. They were kept in the solution for 24 h while the solution was stirred at a rotation speed of 150 rpm.

### 2.2. Characterization of ZnO Thin Films

The morphology of the electrodeposited ZnO films was investigated with a scanning electron microscope (SEM) JSM 6700F (JEOL, Akishima, Tokyo, Japan). Profilometry was used to determine the thickness of ZnO films, using a Veeco Instruments Dektak 6M Stylus Profiler (Veeco Instruments Inc., Dornach Munich, Germany). The thickness of ZnO films on ITO-PET substrates that were fabricated in the miniplant setup could not be determined by profilometry because of the adjacency of the PSAs to the film edges and the impossibility to scratch films on plastic foils. The thickness of these films was derived from SEM images instead.

The adsorption isotherm of Kr at about 77 K was determined by an ASAP 2010 volumetric adsorption unit (Micromeritics), liquid nitrogen being used as a coolant. The sample to be measured was placed in a tailor-made adsorption cell. Prior to the adsorption experiment, the sample was outgassed for 48 h at 150 °C. The specific surface area was determined by the BET method using the molecular cross-sectional area of Kr of 0.21 nm^2^ and the saturation pressure of solid Kr of about 1.6 Torr. Details are given in [38].

### 2.3. Device Fabrication

#### 2.3.1. Fabrication of Small-Scaled Flexible DSSCs

The photo-anodes of small-scaled DSSCs featured ZnO thin films consisting of a dense ZnO film and a porous ZnO film. Both, the photo-anodes and the counter electrodes were based on ITO-PET foil substrates LR15. The counter electrodes were prepared by platinum sputtering (Cressington Scientific Instruments Inc. sputter coater 108auto, Watford, UK) for 120 s at a current of 30 mA and an argon pressure of 0.1 mbar.

After drying for 2 h at 100 °C, the ZnO films were sensitized for 1 h in a solution of the dye D149 (*c* = 0.5 mmol·L^−1^, Mitsubishi Paper Mills Ltd., Tokyo, Japan) and the additive cholic acid (*c* = 1 mmol·L^−1^, Carl Roth, ≥99.0%) in a 1:1 mixture of acetonitrile (Carl Roth, ≥99.9%, ≤10 ppm H_2_O) and tert-butanol (ABCR, ≥99.9%). The sensitized ZnO films were subsequently rinsed with acetonitrile and dried for 1 h at 80 °C. PSAs (tesa^®^ 61562) with a circular cut-out, defining the active area to 1.33 cm^2^, were used as spacers and, at the same time, as sealants between the two electrodes. The assembled cells were filled with electrolyte using the backfilling method through a hole in the counter electrode. A special syringe (Solaronix Vac’n’Fill Syringe, Aubonne, Switzerland) was used for this procedure. Afterwards the hole was sealed with PSA. The electrolyte was a solution of tetrapropylammonium iodide (*c* = 1 mol·L^−1^, ≥98%Sigma-Aldrich, St. Louis, MO, USA) and iodine (*c* = 0.1 mol·L^−1^, Sigma-Aldrich, ≥99.999%) in a 1:4 mixture of acetonitrile and ethylene carbonate (≥99.0%, Acros Organics, Geel, Belgium).

#### 2.3.2. Fabrication of Up-Scaled Flexible DSSC Modules

The procedure for the assembly of up-scaled DSSC modules was analogous to that of small-scaled DSSCs, with the following differences: The platinum coating of the counter electrodes was performed twice with different orientations of the substrates to achieve a coating as homogeneous as possible on this comparatively large area. The PSAs used for sealing the cells were those already present on the photo-anode substrates during ZnO deposition (see Section 2.1.3). To fill the individual cells with electrolyte, the counter electrodes contained two holes per segment. With a syringe the electrolyte was pushed through one hole into the segment, until it emerged from the second hole.

### 2.4. Photovoltaic Characterization

#### 2.4.1. I-V Characteristics

*I*-*V* characteristics of the DSSC modules were recorded under real sunlight. The sweep rate was 50 mV·s^−1^. A pyranometer (Kipp & Zonen CMP 21, Delft, The Netherlands) was used to simultaneously measure the incident light intensity. Directly after the measurements the module temperatures were determined using a contact thermometer (Testo 905-T2, Sparta, USA). Besides the characterization under real sunlight, the small-scaled DSSCs were also characterized under simulated sunlight with an irradiance of 1000 W·m^−2^. A Xenon arc lamp (Oriel Instruments 66901/69911, Newport Spectra Physics, Darmstadt, Germany) equipped with filters to generate AM 1.5D conditions served as the light source. The light intensity was adjusted with a thermopile (Kipp & Zonen CA2, Delft, The Netherlands). The reported current densities and conversion efficiencies refer to the photoactive area of the solar cells.

#### 2.4.2. Electrochemical Impedance Spectroscopy (EIS)

Electrochemical impedance spectra were recorded under illumination with simulated sunlight (AM 1.5D conditions) of about 550 W·m^−2^ being the highest light intensity at which an almost homogeneous illumination of the DSSC modules could be maintained using the given light source. The measurements were performed with an electrochemical workstation IM6e (ZAHNER-Elektrik, Kronach, Germany). For the analysis of the data the software ZVIEW (Version 3.3b) from Scribner Associates Inc. (Southern Pines, NC, USA) was used.

## 3. Results and Discussion

### 3.1. Electrodeposition of ZnO Films

#### 3.1.1. Adaption of the ZnO Electrodeposition Process to the Miniplant Setup

Effects associated to the up-scaling of the ZnO electrodeposition process required the following adjustments of the deposition method:

(1) The increased dimensions of the setup in comparison to the RDE setup cause increased ohmic voltage drops when a deposition voltage is applied. Predominantly this applies to the substrates with their relatively high ohmic resistances. Without modifications of the substrates these voltage drops can result in considerably more positive potentials at the centers of the deposition areas compared to their edges. Consequently the ZnO growth in the centers is slower than at the edges, giving non-uniform films. If a more negative voltage is applied to obtain an adequate film growth in the centers, this can cause the deposition of elemental zinc at the edges.

To reduce the described voltage drops on the substrates and thereby permit the deposition of uniform ZnO films, the substrates were divided into several smaller segments surrounded by conductive silver grids. On the FTO-glass substrates this resulted in three deposition segments with a cumulative area of 38.88 cm^2^.

Since the sheet resistances of the ITO-PET substrates are higher than those of the FTO-glass substrates, a pattern with 12 smaller deposition segments, giving a cumulative deposition area of 34.56 cm^2^ was necessary for these substrates. The latter layout follows simulation results by Zhang et al. [39] for optimized DSSC module performances. The limitation of the series resistance of a module is crucial to reduce the ohmic power dissipation, as is discussed in Section 3.2.

(2) When the electrodeposition was always performed at the same potential vs. the reference electrode as is usually the case in the RDE setup, this actually resulted in varying film properties. Equal deposition conditions delivered either films of ZnO or films containing elemental Zn. It was observed that prior to the deposition the rest potentials of the substrates drift towards constant values, which, however, differ from substrate to substrate. For example in the miniplant setup with an FTO-glass substrate the rest potential drifted in one case from about 30 mV vs. Ag/AgCl to about 60 mV vs. Ag/AgCl during a time span of about 20 min. Although the drift velocity decreased, a constant value even was not reached after 20 min. In contrast, in the RDE setup the rest potential drifts by about 60–70 mV in the first 10 min, reaching a constant value of about 200 mV vs. Ag/AgCl.

The observed drifts indicate changing properties of the substrate surface, caused for instance by adsorption processes. In the miniplant setup this process appears to occur on a longer time scale than in the RDE setup. Consequently the conditions present at the beginning of the deposition have a significant influence on the deposition process. To take into account the observed on-going drift of the rest potential, electrodeposition in the miniplant setup was performed potentiostatically vs. the rest potential of the substrate after an equilibration time of about 20 min. The rest potential was measured for 5 s immediately before the start of the deposition. Only by this procedure was a high reproducibility of the deposition process possible, especially on ITO-PET substrates.

The applied deposition voltage depended on the substrate type and was adapted to allow the highest deposition current possible without formation of elemental zinc. For the deposition on FTO-glass substrates a voltage of −1.0 V vs. the rest potential vs. Ag/AgCl was applied. With ITO-PET substrates OC^TM^50 and LR15, the deposition voltage was set to −0.93 V and −0.95 V vs. the rest potential vs. Ag/AgCl, respectively. The rest potentials in the miniplant setup, on which the deposition voltages relied, were in the range between −80 mV to 10 mV vs. Ag/AgCl for FTO-glass substrates and in the range between −60 mV to 35 mV vs. Ag/AgCl for ITO-PET substrates.

**Figure 2:**
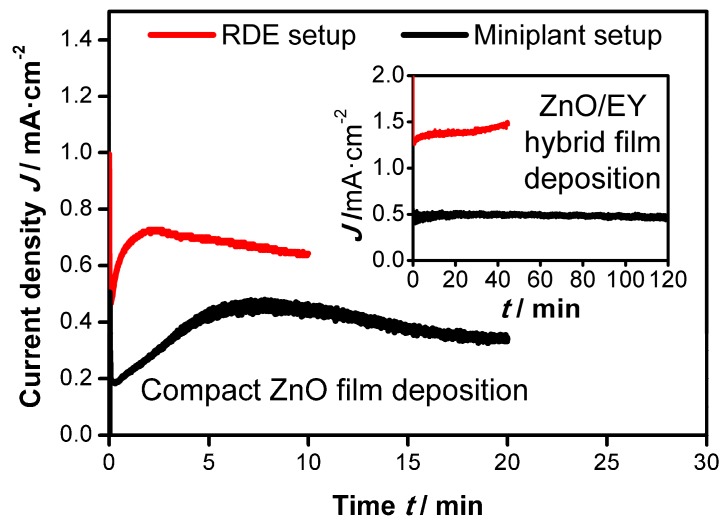
Comparison of current-time behavior between rotating disc electrode (RDE) setup (red lines, at −0.91 V vs. Ag/AgCl) and miniplant setup (black lines, at −1.0 V vs. the rest potential vs. Ag/AgCl) during the electrodeposition on FTO-glass substrates: Deposition of compact ZnO films (full scale) and subsequent deposition of ZnO/EY hybrid films (inset).

(3) In the miniplant setup lower current densities than in the RDE setup were obtained. When for example ZnO/EY hybrid films were deposited, current densities of up to 1.5 mA·cm^−2^ were observed in the RDE setup, while the current densities in the miniplant setup were limited to 0.5 to 0.8 mA·cm^−2^, depending on the substrate layout and the sheet resistance (compare Figure 2). As lower current densities cause a slower film growth, longer deposition times are needed in the miniplant setup to obtain ZnO thicknesses comparable to those obtained in the RDE setup.

Probably the current density in the miniplant setup is decreased by the higher ohmic resistances due to the enlarged dimensions in comparison to the RDE setup. This can include resistances of cables, substrates, and of the electrolyte solution. As mentioned before, the substrate resistance constitutes a major obstacle for the ZnO deposition that was improved by conductive silver paths. The resistance of the electrolyte solution is expected to be higher than in the RDE setup because of the higher distance between working electrode and counter electrode. The contribution of the cable resistance to the reduced current density in the miniplant setup is expected to be small in comparison to the two other quoted factors.

The convection strength in the solution certainly differs between the RDE and the miniplant setup because of the different dimensions and mixing concepts. This probably has an influence on the reagent transport from and to the substrate and consequently the rate of film growth, too. An optimization of the paddle movement frequency might lead to a faster film growth and shorter deposition times in the miniplant setup.

The reproducibility of the deposition of ZnO/EY hybrid films in the miniplant setup was generally sufficient. On all substrate types the film thickness correlated to the transferred charge per area, indicating constant deposition efficiency. The deposition rate of the hybrid films amounts to about 1.4 µm·C^−1^·cm^−2^, which is similar to deposition rates reported for small-area depositions in RDE setups [40].

#### 3.1.2. Electrodeposition of ZnO Films on FTO-Glass Substrates

Depositions on FTO-glass substrates were first used to demonstrate that the miniplant setup is suitable to produce ZnO films comparable to those obtained on small substrates in an RDE setup. In Figure 2 the current-time curves are shown for both deposition setups. During the deposition of compact ZnO films in both setups an initial increase of the deposition current is observed, which can be attributed to the three-dimensional growth of the individual ZnO crystals. The maximum current density is obtained when the crystals start merging, leading to a transition from three-dimensional to one-dimensional growth and a decrease in the current density [27,33].

When ZnO/EY hybrid films are deposited (inset in Figure 2) on top of the compact ZnO layer, the current density remains nearly constant over time, meaning that the one-dimensional film growth seen towards the end of the deposition of the compact ZnO layer is continued. Due to the catalytic effect of EY on the oxygen reduction the current densities are increased in comparison to the deposition of compact ZnO films in both setups [24,30].

**Figure 3:**
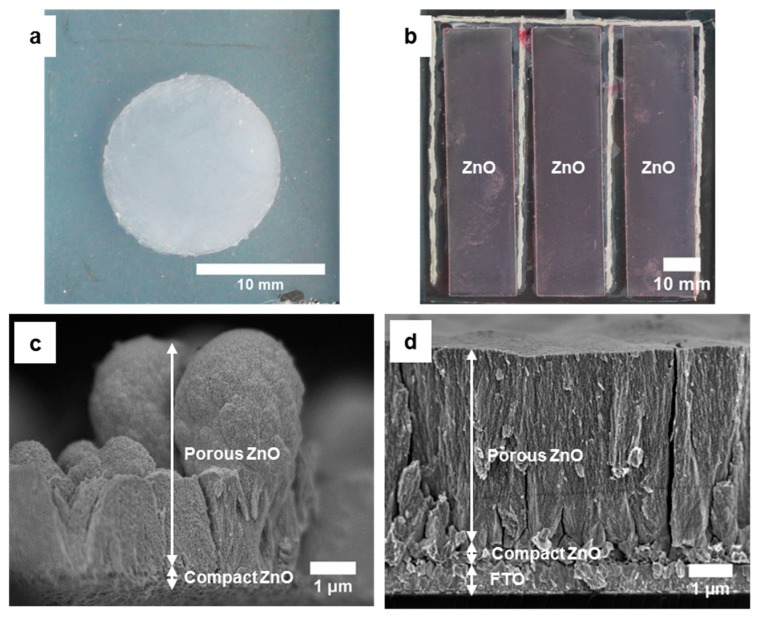

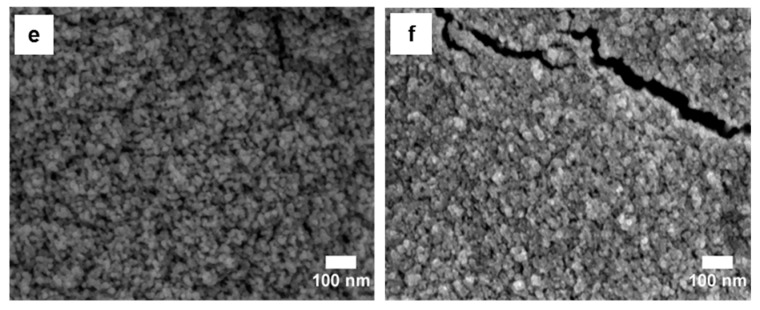
Morphology of ZnO films electrodeposited on FTO-glass substrates in the RDE setup (deposition of compact layer for 10 min and of porous layer for 45 min) and in the miniplant setup (deposition of compact layer for 20 min and of porous layer for 120 min): Photographs of ZnO films after deposition in the RDE setup (**a**) and miniplant setup (**b**); scanning electron microscopy (SEM) cross section images of compact and porous ZnO film deposited in the RDE setup (**c**) and miniplant setup (**d**); SEM top view images of compact and porous ZnO film deposited in the RDE setup (**e**) and miniplant setup (**f**).

The photograph in Figure 3a presents the homogeneous deposition characteristic of the ZnO film which is typical for an RDE deposition process. As seen in the photograph in Figure 3b, the ZnO films deposited on the enlarged substrate in the miniplant setup are also rather homogeneous. The cross section image of the film deposited in the RDE setup (Figure 3c) shows a rather inhomogeneous surface of the film, which also explains the slight increase of the current density over time for the hybrid film in this setup, as some crystals grow higher than others and revert to three-dimensional growth. Irregularities of the substrate surface and the compact ZnO film surface are supposed to influence the hybrid film growth in a way that becomes more distinct with increasing film thickness. The homogeneity of the film thickness from the miniplant setup is proven by the SEM image in Figure 3d. Both, compact ZnO films as well as porous ZnO films show a high degree of substrate coverage. The thickness of the film obtained from the miniplant setup amounts to about 5 µm after deposition of the hybrid film for 120 min. This is less than the thickness of about 7.6 µm of the film from the RDE setup after deposition for 45 min. The slower film growth in the miniplant setup has already been mentioned in Section 3.1.1. A layout with more, but smaller cells, as it was applied on the ITO-PET foil substrates, would probably lead to a lower substrate resistance and therefore a faster film growth. Anyhow, a film thickness of about 5 µm approaches the thickness of films from the RDE setup sufficiently to demonstrate the successful operation of the miniplant setup. The Figure 3c,d document the comparable microscopic growth of the films in both setups. The nanostructure of the porous films can be observed clearly in Figure 3e,f. Films from both setups contain stress cracks in the porous films.

#### 3.1.3. Electrodeposition of ZnO Films on ITO-PET Foils

**Figure 4:**
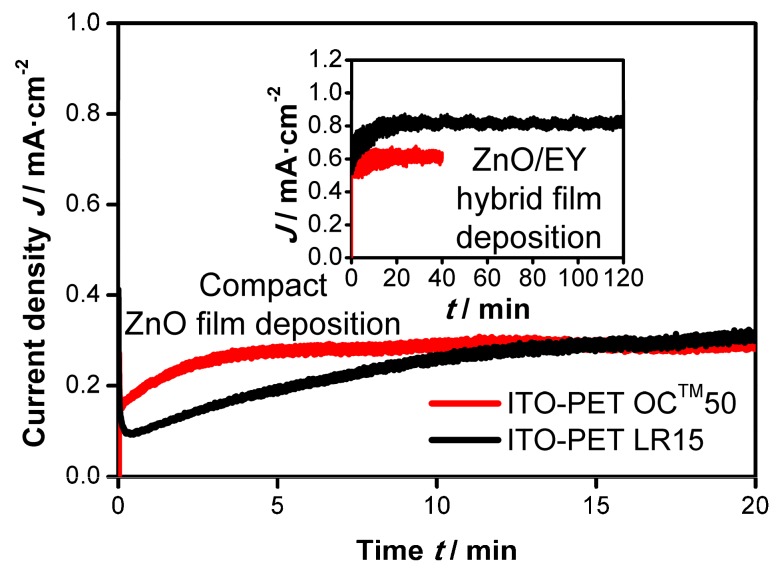
Comparison of current-time behavior during the electrodeposition of compact ZnO films (full scale) and subsequent ZnO/EY hybrid films (inset) on ITO-PET substrates OC™50 (at −0.93 V vs. the rest potential vs. Ag/AgCl) and LR15 (at −0.95 V vs. the rest potential vs. Ag/AgCl) in the miniplant setup.

As mentioned in Section 3.1.1 a modification of the substrate layout was necessary to apply the electrodeposition process on ITO-PET foil substrates. However, despite the change in the layout the current densities during depositions of compact ZnO on these substrates were lower than those on FTO-glass substrates as depicted in Figure 4. No maximum is observed in the current-time curves, indicating that no coalescence of the ZnO crystals is obtained. Considerably longer deposition times or different pre-treatment methods (for example treatment with various reagents, corona discharge, pre-electrolysis etc.) of the substrates might lead to the deposition of dense compact ZnO films on the given ITO-PET substrates.

Again, the current densities during the deposition of ZnO/EY hybrid films on top of the initially deposited ZnO crystals are markedly higher due to the catalytic effect of the EY, as illustrated in the inset of Figure 4. In addition, a comparison with Figure 2 shows that the current density is even higher than that observed during ZnO/EY hybrid film deposition on FTO-glass substrates. This observation leads to two important conclusions: First, it shows the positive effect of the changed layout on the film deposition. Second, it proves that the low current density during the initial ZnO deposition does not occur due to limitation by the electrical resistance of the substrate but probably due to the surface properties of the ITO.

On the substrate type OC™50 the deposition time for the ZnO/EY hybrid films had to be limited to 40 min. In the case of longer deposition times the current density steadily decreased and compact ZnO was deposited on top of the hybrid film. This upper compact film sealed the hybrid film and therefore prevented desorption of the EY from the film and the dye loading. The reason for the transition from ZnO/EY to pure ZnO being deposited is the increasing potential drop caused by the cumulative resistance of the ITO layer (50 Ω·sq^−1^) and the growing ZnO film, which eventually leads to a potential >−0.8 V vs. Ag/AgCl at the electrode surface. In this potential region, EY is not electrochemically reduced, preventing the formation of the nanostructured hybrid film. When a more negative potential than −0.93 V vs. the rest potential vs. Ag/AgCl was used to force the reduction of EY, this resulted in the formation of elemental zinc on the substrate. When the deposition time of the hybrid film at a deposition potential of −0.93 V vs. the rest potential was limited to 40 min, the deposition of the compact ZnO top layer could be avoided.

When ZnO/EY hybrid films are deposited on the substrate type LR15 (again on top of a layer of compact ZnO crystals), the lower sheet resistance (15 Ω·sq^−1^) compared to OC™50 leads to a higher current density (Figure 4). Note that the deposition current on LR15 is actually lower than on OC™50 during the first 15 min of the ZnO bottom layer deposition, which again proves that this process is not limited by the resistance of the substrate, but by the surface properties of the ITO. In this respect, the LR15 material seems to have a disadvantage, which is, however, overcome after 15 min of ZnO deposition. For the deposition of the ZnO/EY layer, the higher conductivity of the LR15 substrate is clearly advantageous, allowing a deposition time of 120 min.

**Figure 5:**
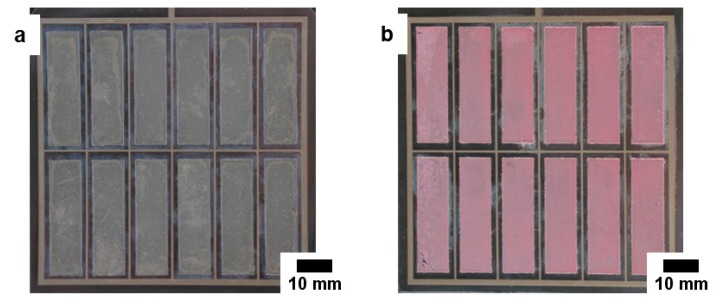

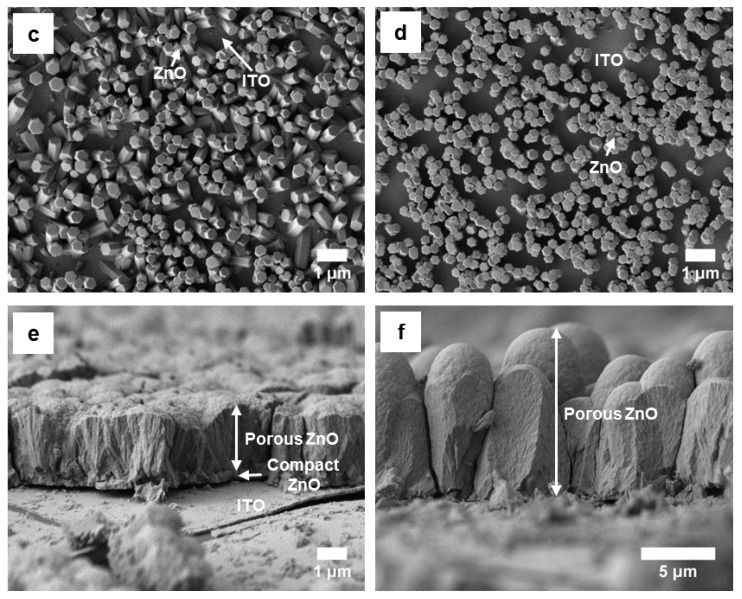
Morphology of ZnO films electrodeposited in the miniplant setup on ITO-PET OC™50 substrates (deposition of compact layer for 20 min and of porous layer for 40 min) and on ITO-PET LR15 substrates (deposition of compact layer for 20 min and of porous layer for 120 min): Photographs of ZnO films after deposition on OC™50 (**a**) and LR15 (**b**); SEM top view images of compact ZnO films deposited on OC™50 (**c**), and LR15 (**d**); SEM cross section images of compact and porous ZnO films deposited on OC™50 (**e**), and LR15 (**f**).

Both, the film deposited on OC™50 and the film deposited on LR15 display a macroscopic homogeneity (Figure 5a,b). As the photography in Figure 5b shows, the film deposited on LR15 exhibits a light pink color, which evidences non-desorbed residues of EY, although no evidence for the deposition of compact ZnO above the ZnO/EY layer could be found. The incomplete dye desorption therefore seems to arise from the high film thickness, making technical optimization of the desorption process seem necessary to remove the EY completely. Due to the entrapment of the eosin Y inside the ZnO pores the dye residues are assumed to have no direct accessibility by the electrolyte, hence they do not contribute significantly to the photocurrent in the assembled solar cells. However, it is very likely that the entrapped eosin Y decreases the ZnO surface area available for adsorption of the photosensitizer dye to some extent.

The SEM images in Figure 5c,d confirm that neither on the OC™50 nor on the LR15 substrate were dense compact ZnO films obtained, as already predicted from the current-time behavior of the electrodeposition.

On the OC™50 substrate ZnO/EY films with a similar morphology to those on FTO-glass substrates were obtained, having a thickness of about 2.5 µm (Figure 5e). Resulting from the longer deposition time the ZnO/EY films deposited on the LR15 substrate have a film thickness of about 9 µm (Figure 5f). As mentioned above no evidence for the deposition of compact ZnO on top of the ZnO/EY film can be found.

**Figure 6:**
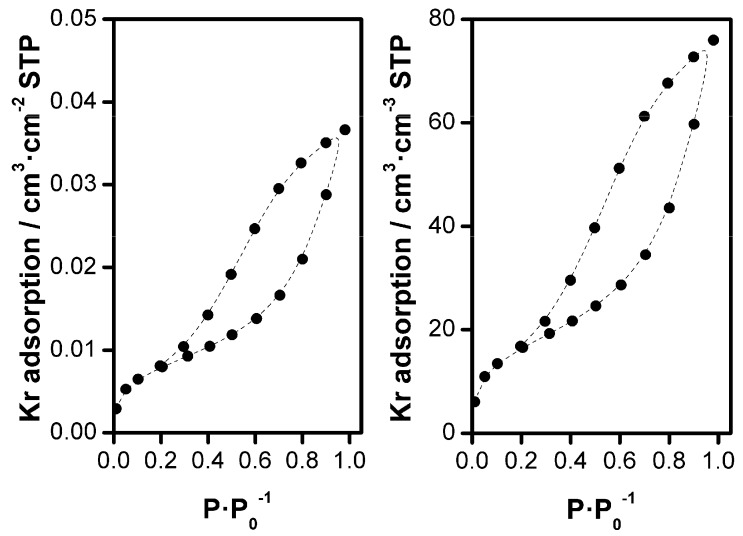
Adsorption isotherms of Kr at about 77 K of an up-scaled porous ZnO film on an ITO-PET substrate LR15 with a thickness of about 4.8 µm. The adsorption is related to 1 cm^2^ of the support and 1 cm^3^ of the ZnO film, respectively.

The porosity of the porous ZnO films obtained from the miniplant setup after desorption of EY was verified exemplary by Kr adsorption measurements with a film deposited on an ITO-PET LR15 substrate. This flexible substrate type was chosen for the adsorption measurement because the ZnO film morphology on ITO or FTO glasses has already proven [35,40] and appeared to be promising for flexible DSSC photo-anodes as well. The resulting isotherms are shown in Figure 6. The well-developed hysteresis proves the presence of pores smaller than about 10 nm in the sample. The specific surface area related to the film area, also called roughness factor (RF), was determined as 373 cm^2^·cm^−2^. The specific surface area related to the film volume amounts to 77 m^2^·cm^−3^. Elsewhere a RF of 220 for a film thickness of 3 µm has been reported [41]. This corresponds to a specific surface area related to the film volume of 73 m^2^·cm^−3,^ and is therefore in good agreement with our value.

Among the porous ZnO films, those prepared on the ITO-PET LR15 substrates were regarded as being most suitable for the use as photo-anodes in modules due to their higher thickness and acceptable porosity and stability. These substrates were therefore used to prepare and test flexible DSSC modules as described in the following section. The presence of a dense compact ZnO bottom layer would be preferable to suppress recombination reactions between substrate and electrolyte but it is not a prerequisite for the operation of a DSSC.

### 3.2. Flexible DSSC Modules

**Figure 7:**
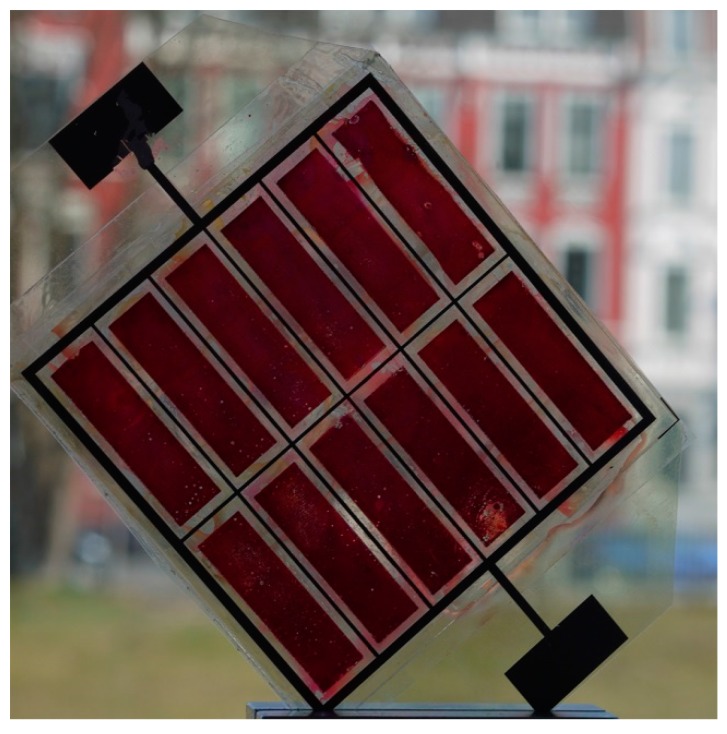
Functional flexible DSSC module with a photoactive area of 34.56 cm^2^. ITO-PET foils are used as substrates for the photo-anode and the counter electrode. The photo-anode contains electrodeposited ZnO films with a thickness of about 9 µm.

**Figure 8:**
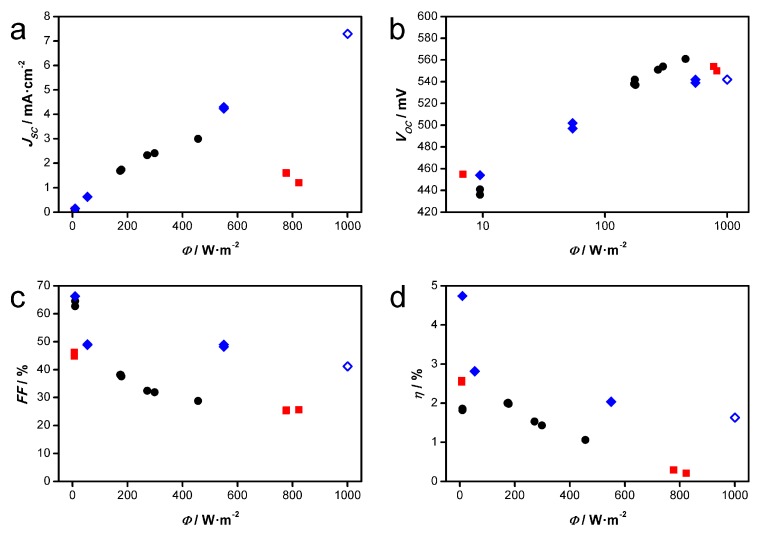
Dependence of photovoltaic parameters on light intensity *ϕ*. (■) Flexible DSSC module “1”. (●) Flexible DSSC module “2”. (♦) Flexible small-scaled DSSC. Open symbol: Measurement performed in solar simulator. All other measurements were performed under real sunlight. (**a**) short circuit current *J_SC_*; (**b**) open circuit voltage *V_OC_*; (**c**) fill factor (FF); (**d**) power conversion efficiency *η*.

A typical module as prepared and tested in this study is depicted in Figure 7. Due to the electrical contact between the active areas on each electrode a parallel connection of the individual cells results. The photovoltaic parameters of two equally prepared modules (flexible DSSC modules “1” and “2”) are depicted in Figure 8 in comparison to a small-scaled DSSC. At low light intensities *ϕ* the *I*-*V* characteristics of the modules exhibit distinctive diode behavior. With increasing *ϕ* values, however, the fill factor (FF) strongly decreases towards 25%, converting the shape of the *I-V* curve to a straight line.

The low fill factor, i.e., the small slope of the *I-V* curve starting from *V_oc_* towards lower voltages, also seems to restrict the short-circuit current density *J_SC_* of the modules at higher *ϕ* values. While a linear increase of *J_SC_* to 7.29 mA cm^−2^ at 1000 W·m^−2^ is seen for a small-scaled flexible DSSC based on a comparable ZnO film (blue symbols in Figure 8), the *J_SC_* seen for the DSSC module “2” (black symbols) starts to deviate from the linear behavior at *ϕ* >200 W·m^−2^. Compared to module “2” with 3.00 mA cm^−2^ at 456 W·m^−2^, module “1” even gives lower *J_SC_* values of 1.60 and 1.20 mA cm^−2^ (red symbols), although *ϕ* was further increased to 777 and 823 W·m^−2^, respectively, which is further discussed below in conjunction with the EIS results. The decrease in both values, *J_SC_* and FF, is the reason for the considerably lower power conversion efficiencies *η* of the modules at higher *ϕ* values, reaching 1.06% at 456 W·m^−2^ for module “2” and only 0.21% at 823 W·m^−2^ for module “1”. *V_oc_* however shows the typical increasing trend with the logarithm of the light intensity for the small-scaled cell as well as the modules. The highest *η* value of 2.58% for module “2” is obtained at *ϕ* = 6.86 W·m^−2^. In contrast, the small-scaled flexible DSSC shows a far less pronounced dependence of FF, *J_SC_* and *η* on the light intensity. While at a low light intensity *ϕ* of 9.48 W·m^−2^ the FF and the *η* of this cell amount to 66.2% and 4.74%, respectively, relatively high values of FF = 41.1% and *η* = 1.63% are retained at *ϕ* = 1000 W·m^−2^.

**Figure 9:**
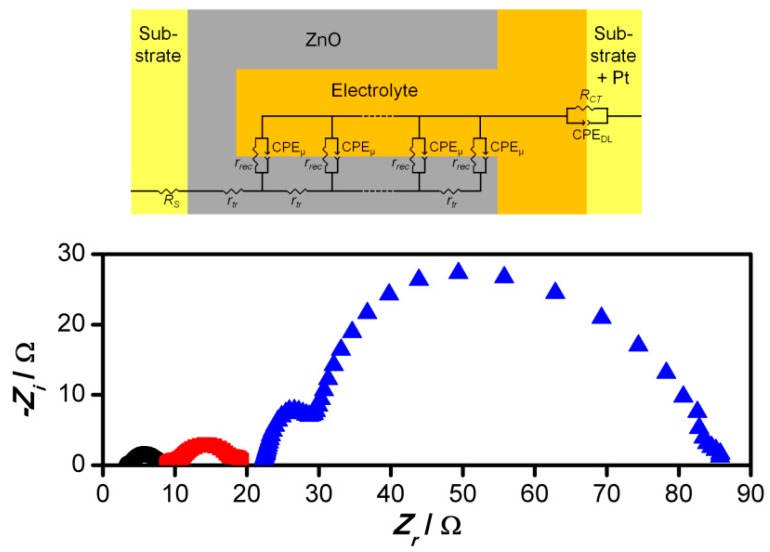
Applied electrochemical impedance spectroscopy (EIS) model for fitting (**top**) and exemplary EIS spectra recorded at *V* = 300 mV (**bottom**). (■) Flexible DSSC module “1”. (●) Flexible DSSC module “2”. (♦) Flexible small-scaled DSSC. *R_S_*: Series resistance. *r_tr_*: Transport resistance. *r_rec_*: Recombination resistance. CPE_µ_: Constant phase element describing the chemical capacitance. *R_CT_*: Charge-transfer resistance. CPE_DL_: Constant phase element describing the double-layer capacitance.

Further investigations of the modules and the small-scaled cell were conducted by EIS. The model used for fitting of the EIS spectra is adapted from a transmission line model suggested by Fabregat-Santiago et al. [42]. A transmission line model is suitable to describe systems that contain porous electrodes like the ZnO films in this study. The elements of the model describe the properties of individual cell components and processes. The applied model and exemplary EIS spectra are shown in Figure 9. At high frequencies—at the left-hand side of the spectra—the impedance is controlled by the series resistance *R_S_* of the cells and by the charge transfer resistance *R_CT_* and the double-layer capacity *C_DL_* of the counter electrode. The latter two form the first semicircle. The second semicircle at lower frequencies is constituted by the transport resistance *r_tr_* and the chemical capacitance *c_µ_* of the ZnO film as well as the recombination resistance *r_rec_* of the ZnO/electrolyte interface. The model uses constant phase elements (CPEs) to describe the capacities. The formula that was used to convert the CPE parameters to equivalent capacities is described in Reference [42] Equation S1.

**Figure 10:**
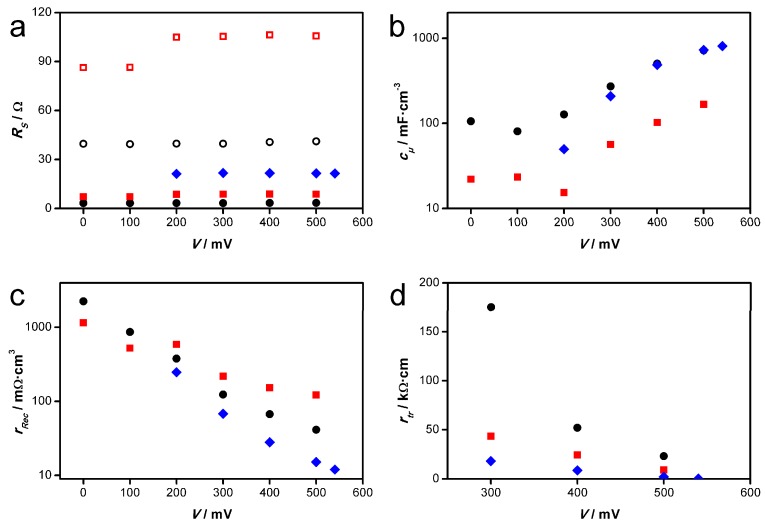
EIS fitting results. (■) Flexible DSSC module “1”. (●) Flexible DSSC module “2”. (♦) Flexible small-scaled DSSC. Open symbols: Estimated series resistances *R_S_* per module cell. (**a**) series resistance *R_S_*; (**b**) chemical capacitance *c_µ_*; (**c**) recombination resistance *r_rec_*; (**d**) transport resistance *r_tr_*.

The fitted parameters are presented in Figure 10. The values for the chemical capacitance *c_µ_* of the ZnO film show no major differences between the modules on the one hand and the small-scaled cell on the other hand. The distinct increase of the chemical capacitances between 100 and 500 mV can be explained by a rise of the quasi-fermi level towards more negative applied potentials, increasing the number of energetic states that can be occupied by electrons. Also the recombination resistances *r_rec_* at the ZnO/electrolyte interface are comparable for the examined modules and the small-scaled cell. The somewhat higher values seen for the modules in a part of the voltage range most probably arise from the fact that some of the pores in the films are not electrochemically accessible as seen in the incomplete eosin Y desorption (see Figure 5 and related discussion). On the other hand, they confirm that the lack of a completely dense compact ZnO layer in these films does not lead to significantly more recombination, since the latter would be expected to decrease the *r_rec_* value. The differences observable in the transport resistances *r_tr_* of the ZnO films are not significant, because the corresponding part of the EIS spectra is difficult to fit. In conclusion, these three parameters related to the porous ZnO film support the assumption that the ZnO films fabricated in the miniplant setup behave similarly to ZnO films from the RDE setup.

Low fill factors in solar cells and modules often result from high series resistances *R_S_*, because the slope of an I-V curve at its onset is limited by the overall cell resistance. Too high series resistances diminish the FF and subsequently the obtainable *J_SC_*, as observed for the modules in this study and also previously in other studies concerning the scale-up of DSSCs [43,44,45,46]. In DSSCs the resistance of the transparent conducting substrate primarily contributes to *R_S_* [42]. With about 8 Ω and 3.3 Ω the series resistances of the modules are lower than that of the small-scaled cell with about 21.5 Ω. However, 12 cells in parallel as present in the modules should only have 1/12 of the resistance of a single cell, meaning that the series resistances per cell in the modules can be estimated at 40 Ω and 96 Ω. At the same current per cell, this leads to a lower FF of the module. Consequently much lower *R_S_* values (<1 Ω) are required to obtain higher fill factors and hence higher conversion efficiencies of the modules under high light intensities.

One reason for the high *R_S_* values of the modules can be seen in the sheet resistance of the used ITO-PET foil substrates (15 Ω·sq^−1^), which is twice as high as that of FTO-glass substrates (7 Ω·sq^−1^). Furthermore, the silver paths may exhibit high contact resistances to the ITO layers, which, however, could not be measured. Also the electrolyte resistance related to the relatively high distance of 50 µm between the two electrodes—necessary to prevent contact between the silver paths of the two electrodes—might have an influence. Besides reduction of the substrate resistances, the connection of the individual cells in series might be helpful to reduce the ohmic power dissipation [5,21]. Furthermore it should be noted that in the case of the modules the charge-transfer resistances *R_CT_* at the counter electrode were relatively high (29.3 and 7.3 Ω·cm^2^, respectively) because for technical reasons the substrates could not be coated homogeneously enough with platinum.

It should be noted at this point that the *R_S_* value of module “1” is even considerably higher than that of module “2”. This explains the lower *J_SC_* value of module “1”, since it leads to a smaller slope of the of *I-V* curve form the photocurrent onset at *V_OC_* towards the *I* axis. Since both modules had the same design and were made from the same materials, the higher *R_S_* value of module “1” is most probably due to corrosion of silver paths by iodine from the electrolyte in this module, indicating that the pastes used for sealing have to be further optimized.

## 4. Conclusions

The electrochemical fabrication of porous ZnO films is an attractive low-temperature method for the preparation of photo-anodes for flexible DSSCs and perovskite solar cells. We demonstrated the scalability of this process from RDE setups for small-scaled films to a technically simplified miniplant setup that was developed for this purpose. The application of conductive silver paths was necessary to limit the voltage drop across the enlarged substrate areas. ZnO electrodeposition was performed successfully on FTO-glass substrates on deposition areas of 38.88 cm^2^. The up-scaled electrochemical deposition process can be applied also on flexible ITO-PET foil substrates, yielding ZnO films with an overall area of 34.56 cm^2^. The properties of the substrates have a strong influence on the success of the deposition. Sheet resistances of the substrates lower than 50 Ω·sq^−1^ are necessary to enable the deposition of hybrid films with thicknesses above 2.5 µm. Furthermore, the deposition of dense compact ZnO films proved to be difficult on the ITO-PET substrates, most probably due to unfavorable surface properties. Under the prerequisite of sufficiently low sheet resistances of the ITO-PET foil substrates (<50 Ω·sq^−1^), ZnO/EY hybrid films with thicknesses of up to 9 µm have been realized. The high porosity of the films after desorption of the EY was proven by means of SEM and Kr sorption. Due to the successful scale-up of the deposition process, also a roll-to-roll process, which would enable high production throughputs, appears realizable.

Operative flexible DSSC modules (*η* = 2.58% at *ϕ* = 6.86 W·m^−2^) proved the suitability of the up-scaled porous ZnO films as DSSC photo-anodes. The analysis of the EIS spectra revealed similar photo-anode properties for up-scaled modules and the small-scaled cells, but also too high series resistances of the modules. These high series resistances cause high ohmic power dissipations and consequently a significant decrease of the fill factor and conversion efficiency of the modules under high light intensities. A technical solution of this problem, for example by the application of different silver pastes and an optimized sealing to avoid corrosion of the silver by the electrolyte, seems possible.
